# LINC00313 promotes the proliferation and inhibits the apoptosis of chondrocytes via regulating miR-525-5p/GDF5 axis

**DOI:** 10.1186/s13018-023-03610-1

**Published:** 2023-02-24

**Authors:** Wen He, Xuchao Lin

**Affiliations:** Department of Orthopaedics, Fuzhou Second Hospital, No. 47, Shangteng Road, Cangshan District, Fuzhou, 350007 Fujian China

**Keywords:** Osteoarthritis, LINC00313, Cartilage injuries, GDF5

## Abstract

**Background:**

The present study aimed to explore the potentials of lncRNA LINC00313 in osteoarthritis (OA).

**Methods:**

qRT-PCR was performed to detect the expression of LINC00313 in OA tissues and cells. CCK-8 and EDU were used to detect cell proliferation. The ELISA test kit was conducted to detect the expression of inflammatory factors. Flow cytometry was used to detect the apoptosis rates. Western blot was applied to measure the protein expression. The luciferase reporter gene test was carried out to verify the relationship between miR-525-5p and LINC00313 or GDF5.

**Results:**

The data showed that the expression of LINC00313 was significantly down-regulated in OA tissues and cells. Functionally, LINC00313 promoted the proliferation of chondrocytes and suppressed the secretion of inflammatory factors and cell apoptosis. Moreover, LINC00313 functioned as a ceRNA to up-regulate the expression of GDF5 via sponging miR-525-5p. Luciferase and RNA pull-down assays further verified the interaction between miR-525-5p and LINC00313 (or GDF5). Moreover, overexpression of miR-525-5p or down-regulated GDF5 degraded the cellular functions of chondrocyte. Rescue experiments showed that the overexpression of miR-525-5p reversed the increase in cell viability and the decrease in pro-inflammatory factors and apoptosis rate mediated by LINC00313. The knockdown of GDF5 reversed the promotion of miR-525-5p knockdown on cell viability and the inhibition of pro-inflammatory factors and apoptosis rate.

**Conclusions:**

LINC00313 inhibited the development of OA through regulating miR-525-5p/GDF5 axis. LncRNA LINC00313 can be used as a potential target for the treatment of OA.

## Introduction

Osteoarthritis (OA) is a common degenerative disease of joints in the middle-aged and elderly people, characterized by progressive destruction of articular cartilage, synovitis and bone hyperplasia [1]. It is clinically manifested as joint pain and dysfunction. At present, there is no radical cure for osteoarthritis. The prevailing strategies for OA are symptomatic treatments and surgery. However, there are side effects for OA, such as increased risk of undergoing arthroplasty [2]. At present, there is still no radical cure for OA [3]. The pathogenesis of osteoarthritis involves multiple factors, multiple links and multiple genes, and the specific mechanism is not very clear. Increasing studies reveal that the damage and destruction of articular cartilage is an important pathological feature of osteoarthritis. Therefore, to unveil the molecular mechanisms underlying articular cartilage damage is of vital essence.

Long noncoding RNA (lncRNA) is about 200 nt ~ 100 kb in length^[[[4]]]^, exists in the cytoplasm to regulate the translation and stability of mRNA and can compete with endogenous RNA (ceRNA) to regulate the distribution of microRNA (miRNA) [5–7]. Previous studies have shown that lncRNA is mainly expressed in human cancers, promoting tumor development, progression and metastasis [8]. LINC00313 is a new type of lncRNA that has been found to regulate the tumorigenesis of papillary thyroid carcinoma (PTC) [9,10]. LINC00313 can accelerate the progression, migration and epithelial–mesenchymal transformation (EMT) of SiHa and Hela cells [11]. Moreover, LINC00313 is down-regulated in OA [12], but the role of LINC00313 in the pathogenesis of osteoarthritis has not been explored.

MiRNAs are a class of small noncoding RNAs that have attracted much attention in recent years. They contain 22–24 nt single-stranded noncoding RNAs that regulate gene expression at the posttranscriptional level and play corresponding biological roles [13]. It has been found that miR-525-5p continues to be dysregulated in various diseases, including glioma and cervical cancer [14,15]. Although there has been a lot of understanding about how miR-525-5p regulates tumorigenesis, the regulation and role in OA are still unknown.

Growth and differentiation factor 5 (GDF-5), also known as cartilage morphogenetic protein 1, belongs to the branch of the transforming growth factor beta superfamily. It is closely related to bone morphogenetic protein [16]. Studies have pointed out that GDF-5 plays an important regulatory role in cell differentiation, human growth and development, and repair of damaged cartilage and bone [17]. It can also treat temporomandibular joint osteoarthritis by affecting the expression of TMJ-OA-related molecules in chondrocytes induced by IL-6 and reducing the expression of matrix metallopeptidase 13 (MMP13) [18].

This study investigated the expression of LINC00313 and miR-525-5p between OA tissues/cells and normal tissues/cells, and explored the biological functions of LINC00313 and miR-525-5p in proliferation, apoptosis and expression of inflammatory factors. At the same time, we studied the relationship between LINC00313 and its target genes miR-525-5p and GDF5. Overall, our results indicate that LINC00313 accelerates the progression of OA by inhibiting the miR-525-5p/GDF5 axis.

## Methods and materials

### Patients and specimens

OA cartilage tissues were taken from the knee joints of OA patients (18) who had undergone total knee arthroplasty. Normal cartilage tissue was taken from the joints of femoral neck fracture patients (11 patients) without OA or rheumatoid arthritis. The study was approved by the Human Ethics Committee of Fuzhou Second Hospital.

### Cell culture and transfection

Human normal chondrocyte C28/I2 cell line was purchased from the Cell Bank of the Chinese Academy of Sciences (Shanghai, China). C28/I2 cells were incubated in RPMI-1640 containing 10% FBS at 37 °C and 5% CO_2_.

Small interfering RNA (siRNA) targeting GDF5 (si-GDF5), miR-525-5p mimics, LINC00313 overexpression vector and negative controls were bought from GenePharma (Shanghai, China). All plasmids were transfected into cells using Lipofectamine 2000 (Invitrogen, Carlsbad, CA, USA).

### CCK-8 assay

After the cells were transfected, the cells were planted in a 96-well plate according to the cell volume of 1 × 10^3^/well, 3 replicate wells were set up, and cells were placed in the incubator for 24 h; 10 μl of CCK-8 solution was added to each well (Dojindo, Japan), incubated in incubator for 2 h and then used a microplate reader to detect the optical density (OD) value at 450 nm to calculate the cell proliferation inhibition rate. Cell proliferation inhibition rate (%) = (control group OD value-knockdown group OD value (/ control group OD value-blank group OD value) × 100%.

### EdU

After transfection, cells incubated then with EdU (20 mmol/L) for 2 h. The cells were then fixed with 4% paraformaldehyde for 20 min at room temperature. The EdU-positive cells were analyzed in different treatment groups.

### qRT-PCR

TRIzol reagent (Invitrogen) was applied to extract and purify total RNA from tissues and cells. Then, RNA was recovered into cDNA using PrimeScript RT kit (Takara, Dalian, China), and amplification experiment was performed using SYBR Premix ExTaqII kit (Takara). Increasing conditions: 95 °C for 10 min (45 cycles), 95 °C for 15 s, 60 °C for 20 s and 72 °C for 20 s. The gene expression level was calculated by the 2^−ΔΔ^CT method.

### Flow cytometry

The cells (1 × 10^6^ cells) were collected and incubated with the V-FITC and PI in the FITC Annexin V Apoptosis Detection Kit (BD Biosciences, Franklin, NJ, USA) at 4 °C for 15 min. Then, the cells were washed 3 times with pre-chilled PBS and suspended in buffer for the next analysis.

### Luciferase assay

The LINC00313 and GDF5 fragment sequences containing wide-type or mutant-type miR-525-5p binding sites were cloned into pGL3-Basic luciferase vector (Promega, Madison, WI, USA) to generate LINC00313-WT, LINC00313-Mut, GDF5-WT and GDF5-Mut. In addition, the luciferase vector was transfected into the cells together with miR-525-5p mimic or miR-NC. A dual luciferase detection system (Promega) was used to assess relative luciferase activity 48 h after transfection.

### Western blot

The cells were harvested, and the lysis buffer was used to extract the protein. After the protein concentration was determined by the BCA method, 50 μg of protein were separated by a 10% SDS-PAGE gel and then transferred to the PVDF membrane. Block the membrane in 5% milk for 1 h; then, incubate with the primary antibody overnight at 4 °C. The membrane was washed 3 times with TBST, and the secondary antibody was incubated for 1 h at room temperature. Finally, an enhanced chemiluminescence solution (Pierce; Thermo Fisher Scientific) was used for protein signal detection.

### Statistical analysis

SPSS 21.0 software was used to input data. Data were expressed as mean ± standard deviation (SD). Analysis of variance was used for comparison between multiple groups, and LSD-t test was used for comparison between pairs. P < 0.05 indicates that the difference is statistically significant.

## Results

### LINC00313 was down-regulated in OA tissue and chondrocytes induced by LPS

**Fig. 1:**
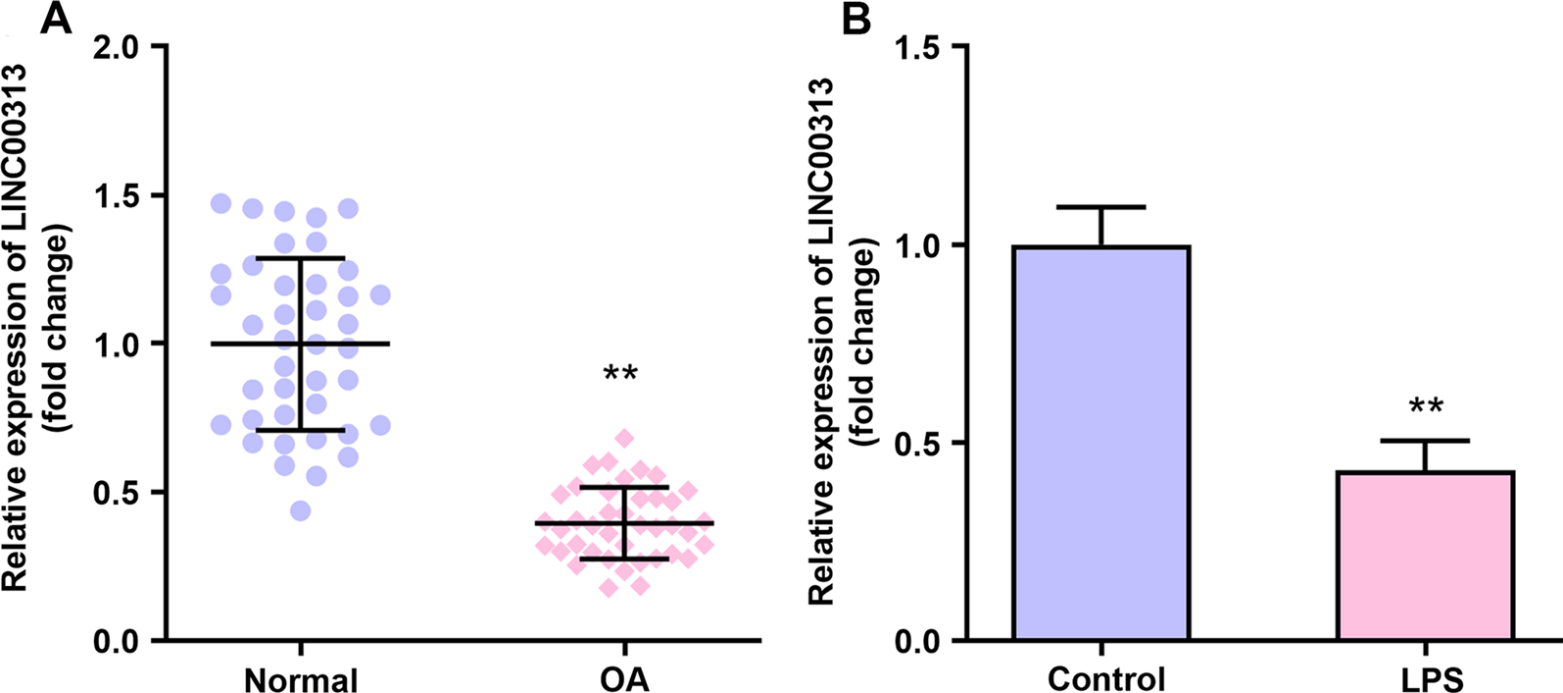
LINC00313 was down-regulated in OA tissue and LPS-induced chondrocytes. **A**: Relative expression of LINC00313 in OA organization. **B**: Relative expression of LINC00313 in chondrocytes induced by LPS. *P < 0.05, **P < 0.001

We studied the expression of LINC00313 in 11 healthy tissue donors and 18 OA patients. qRT-PCR showed that the expression level of LINC00313 in OA tissues was significantly lower than that of normal tissues (Fig. 1A). In LPS-treated C28/I2 cells, the expression level of LINC00313 in OA tissues was significantly lower than that of normal cells (Fig. 1B). Therefore, the results showed that aberrant down-regulated LINC00313 is associated with OA.

### Effect of LINC00313 on C28/I2 cells induced by LPS

**Fig. 2:**
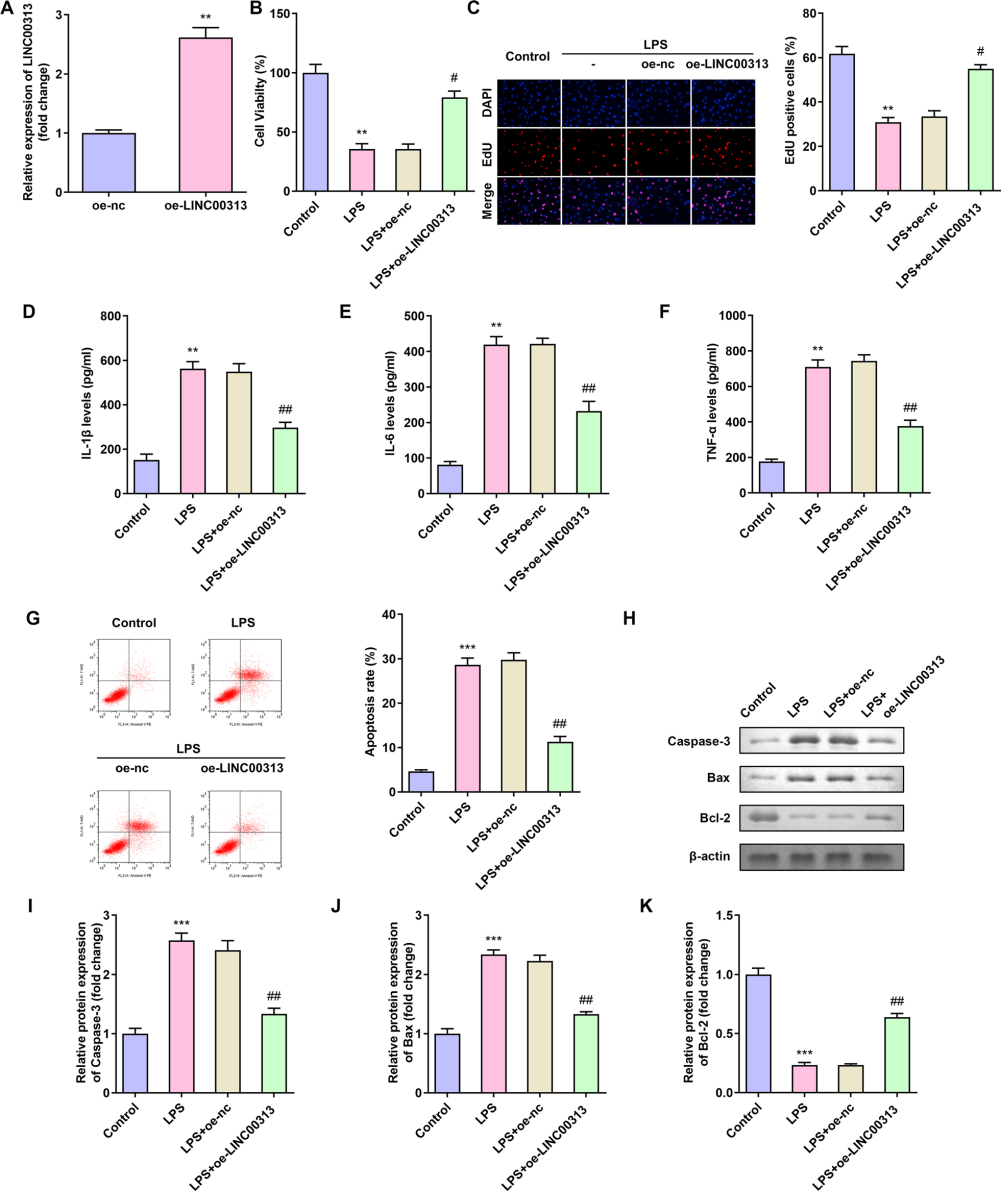
LINC00313 regulated chondrocyte damage induced by LPS. **A**: Transfection effect of overexpression of LINC00313 on C28/I2 cells. **B**–**C**: Overexpression of LINC00313 eliminated the effect of LPS on C28/I2 cell viability. **D–F**: Overexpression of LINC00313 reduced the expression of IL-1β, IL-6 and TNF-α in C28/I2 cells treated with LPS. **G–K**: Overexpression of LINC00313 reversed the effect of LPS on C28/I2 cell apoptosis. *P < 0.05, **P < 0.001; ^#^*P* < 0.05, ^##^*P* < 0.001

We have selected LPS-induced C28/I2 cells as a cell model of OA to determine the biological function of LINC00313 in OA. The expression of LINC00313 in C28/I2 cells was increased after the overexpression of LINC00313 detected by qRT-PCR (Fig. 2A). The results of EdU and CCK-8 experiments showed that LPS significantly reduced the viability of C28/I2 cells, while overexpression of LINC00313 eliminated this effect (Fig. 2B, C). ELISA test results showed that overexpression of LINC00313 significantly reduced the expression of pro-inflammatory cytokines (IL-1β, IL-6 and TNF-α) in C28/I2 cells treated with LPS (Fig. 2D, E, F). Flow cytometry and WB experiments showed that LPS significantly increased C28/I2 cell apoptosis (Fig. 2G) and increased apoptosis-related proteins, while overexpression of LINC00313 reversed this effect (Fig. 2H, I, J, K). These findings indicated that overexpression of LINC00313 promoted cell proliferation in OA and inhibit cell inflammatory factor secretion and apoptosis.

### LINC00313 is the direct target gene of miR-525-5p

**Fig. 3:**
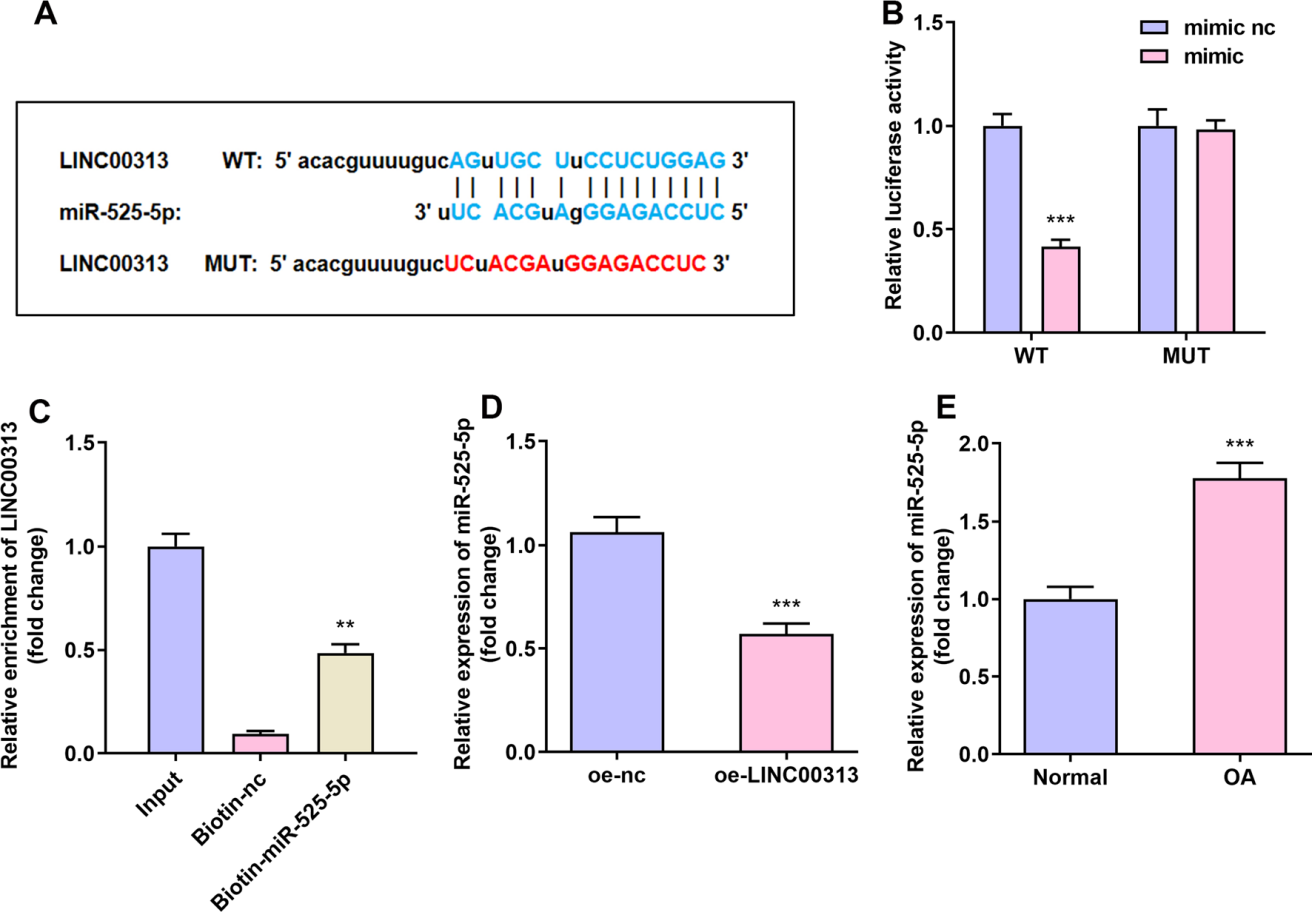
LINC00313 is the direct target gene of miR-525-5p. **A**: Use online prediction software to predict the targeting relationship between LINC00313 and miR-525-5p. **B**: Luciferase activity experiment was used to verify the targeting relationship between LINC00313 and miR-525-5p. **C**: Relative mRNA enrichment of LINC00313 captured by biotin-miR-525-5p. **D**: miR-525-5p was reduced in C28/I2 cells overexpressing LINC00313. **E**: miR-525-5p was elevated in OA tissue. *P < 0.05, **P < 0.01, ***P < 0.001

Through bioinformatics prediction, it was found that LINC00313 contains the binding site of miR-525-5p (Fig. 3A). Luciferase reporter gene detection showed that miR-525-5p mimic reduced the relative luciferase activity of LINC00313-WT group (Fig. 3B). In addition, compared with the biotin-NC group, the LINC00313 mRNA captured by biotin-miR-525-5p was significantly higher (Fig. 3C). The expression of miR-525-5p was significantly reduced by LINC00313 overexpression plasmids (Fig. 3D). The expression of miR-525-5p increased in OA tissues (Fig. 3E). The above results showed LINC00313 directly targeted by miR-525-5p.

### Overexpression of miR-525-5p reverses the effect of LINC00313 in C28/I2 cells

**Fig. 4:**
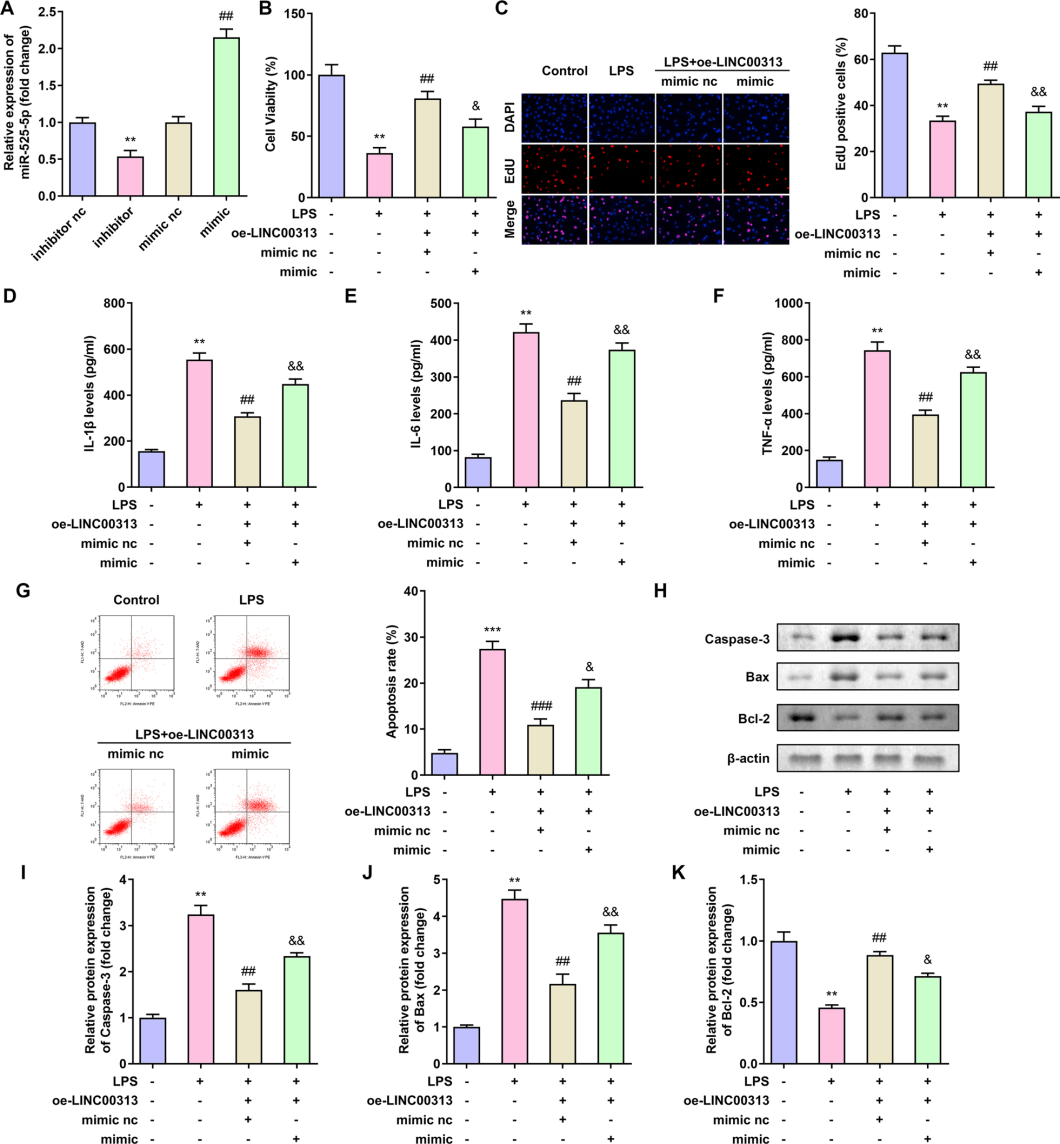
Overexpression of miR-525-5p reversed the effect of LINC00313 in C28/I2 cells. **A**: Transfection effect of miR-525-5p inhibitor and overexpression miR-525-5p on C28/I2 cells. **B**–**C**: Overexpression of miR-525-5p reduced the effect of LINC00313 on C28/I2 cell viability. **D**–**F**: Overexpression of miR-525-5p reduced the effect of LINC00313 on the expression of IL-1β, IL-6 and TNF-α in C28/I2 cells. **G**–**K**: Overexpression of miR-525-5p reversed the effect of LINC00313 on C28/I2 cell apoptosis. *P < 0.05, **P < 0.01, ***P < 0.001; ^#^*P* < 0.05, ^##^*P* < 0.01, ^###^*P* < 0.01

In order to further study the regulatory network of LINC00313 and miR-525-5p, miR-525-5p and LINC00313 were co-transfected into C28/I2 cells. The transfection efficiency of C28/I2 cells treated with miR-525-5p inhibitor and transfected with miR-525-5p mimic was confirmed (Fig. 4A). Overexpression of LINC00313 promotes cell proliferation, while up-regulation of miR-525-5p reduces cell proliferation (Fig. 4B, C). Overexpression of LINC00313 reduced the expression of inflammatory factors IL-1β, IL-6 and TNF-α, while up-regulation of miR-525-5p reversed the expression of inflammatory factors IL-1β, IL-6 and TNF-α (Fig. 4D, E, F). Overexpression of LINC00313 reduced the rate of apoptosis (Fig. 4G), the expression of apoptotic proteins Bax and Caspase-3, and increased the expression of anti-apoptotic protein Bcl-2 (Fig. 4H, I, J, K), while the up-regulation of miR-525-5p reversed the effect of LINC00313. These findings indicated that the up-regulated miR-525-5p reversed the effect of LINC00313 on C28/I2.

### GDF5 is the target of miR-525-5p

**Fig. 5:**
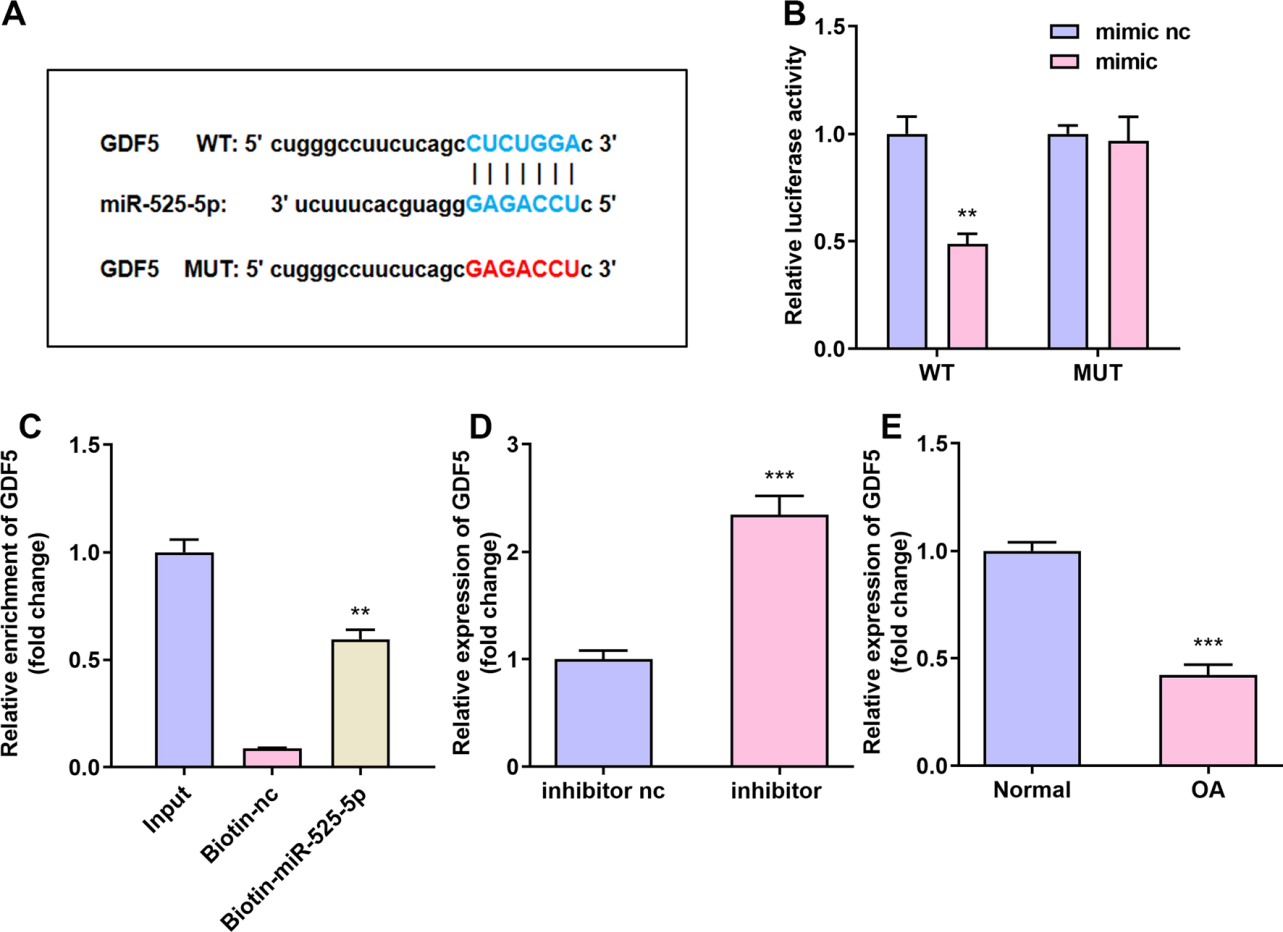
miR-525-5p was the target of GDF5. **A**: Use online prediction software to predict the targeting relationship between GDF5 and miR-525-5p. **B**: Luciferase activity experiment was used to verify the targeting relationship between GDF5 and miR-525-5p. **C**: Relative mRNA enrichment of GDF5 captured by biotin-miR-525-5p. **D**: GDF5 is elevated in C28/I2 cells that inhibit miR-525-5p. **E**: GDF5 is reduced in OA organizations. *P < 0.05, **P < 0.01, ***P < 0.001

We explored the potential targets of miR-525-5p through bioinformatics analysis. Our results indicate that GDF5 may be targeted by miR-525-5p (FigureA). The results of the luciferase reporter gene test showed that miR-525-5p mimics significantly inhibited the luciferase activity of the GDF5-WT group (Fig. 5B). In addition, compared with the biotin-NC group, the GDF5 mRNA captured by biotin-miR-525-5p was significantly higher (Fig. 5C). We found the miR-525-5p expression was significantly increased after miR-525-5p was inhibited in C28/I2 cells by using qRT-PCR (Fig. 5D). The expression of miR-525-5p in OA tissues decreased (Fig. 5E).

### Inhibition of GDF5 reversed the effect of miR-525-5p in C28/I2 cells

**Fig. 6:**
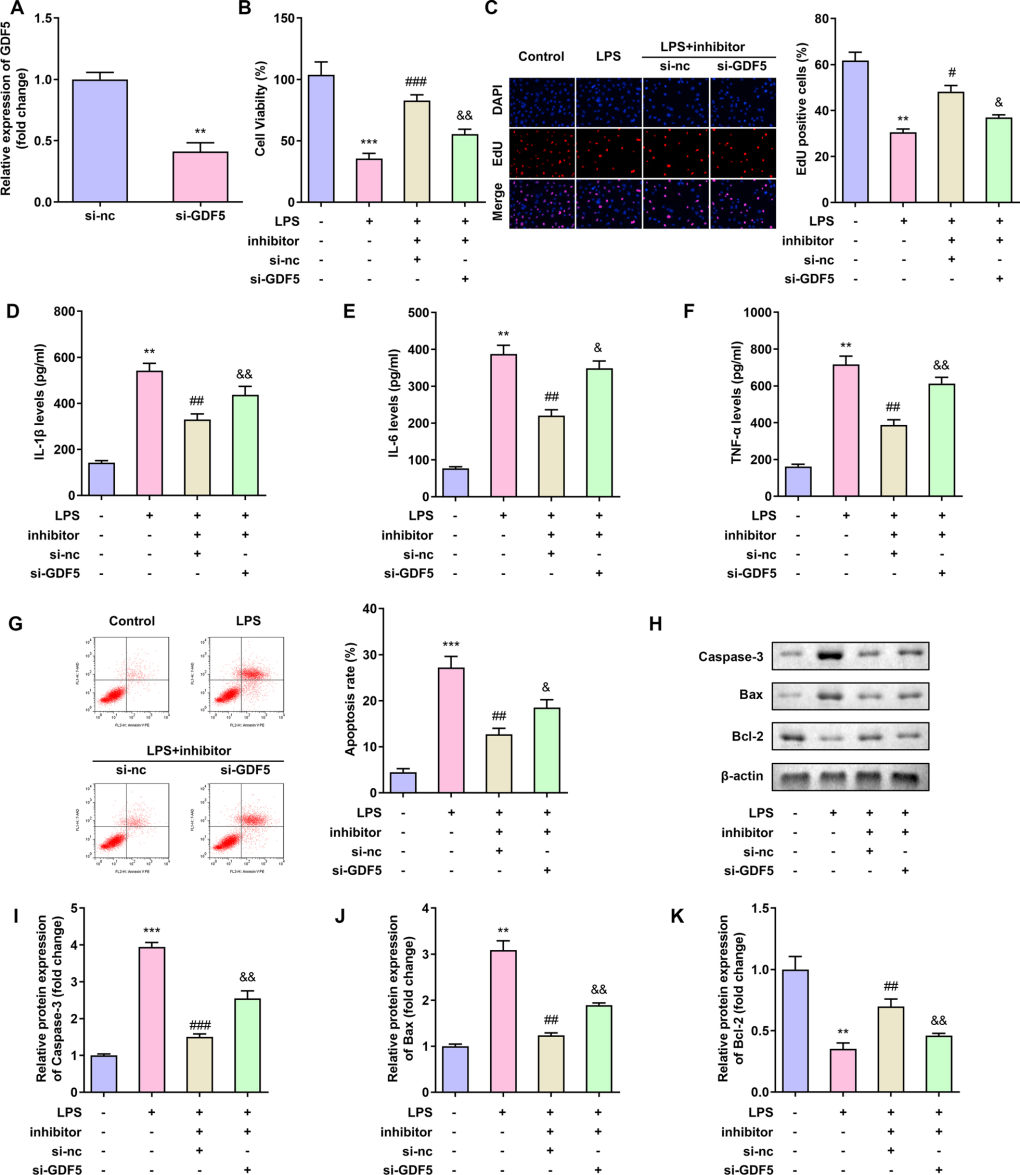
Inhibition of GDF5 reversed the effect of miR-525-5p in C28/I2 cells. **A**: Transfection effect of transfected si-GDF5 in C28/I2 cells. **B**–**C**: Inhibition of GDF5 reversed the effect of miR-525-5p on C28/I2 cell viability. **D–F**: Inhibition of GDF5 reduced the effect of miR-525-5p on the expression of IL-1β, IL-6 and TNF-α in C28/I2 cells. **G**–**K**: Inhibition of GDF5 reduced the effect of miR-525-5p on C28/I2 cell apoptosis. *P < 0.05, **P < 0.01, ***P < 0.001; ^#^*P* < 0.05, ^##^*P* < 0.01, ^###^*P* < 0.01

To further confirm the miR-525-5p/GDF5 axis in OA, we measured the expression of GDF5 in C28/I2 cells transfected with si-GDF5. The expression of GDF5 in C28/I2 cells decreased after the transfection with si-GDF5 (Fig. 6A). Inhibition of miR-525-5p increases cell proliferation, while knockdown of GDF5 reduced cell viability (Fig. 6B, C). Inhibition of miR-525-5p reduced the expression of inflammatory factors IL-1β, IL-6 and TNF-α, while knockdown of GDF5 reversed the expression of inflammatory factors IL-1β, IL-6 and TNF-α (Fig. 6D, E, F). Inhibition of miR-525-5p reduced the rate of apoptosis (Fig. 6G), the expression of apoptotic proteins Bax, Caspase-3 and increased the expression of anti-apoptotic protein Bcl-2 (Fig. 6H, I, J, K), while knockdown of GDF5 reversed the effect of LINC00313. These findings indicate that miR-525-5p/GDF5 can be used to repair bone and articular cartilage damage.

## Discussion

To our knowledge, this is the first study to investigate the potentials of LINC00313 in OA. We found that LINC00313 was down-regulated in OA tissues. Overexpression of LINC00313 promoted the proliferation and inhibited the apoptosis of chondrocytes via miR-525-5p/GDF5 axis. Thence, LINC00313 may play a protective role in OA.

Competitive binding of mRNA and miRNA is the main way for lncRNA to regulate biological functions [19]. lncRNA FOXD2-AS1 is down-regulated in OA patients; the overexpression of FOXD2-AS1 promotes chondrocyte proliferation and inhibits the development of OA through the miR-27a/TLR4 axis [20]. In addition, lncRNA DANCR promoted OA chondrocytes proliferated through the miR-216a/JAK2 axis and the miR-577 / Sphk273 axis [21]. LINC00313 is a new type of lncRNA, and its up-regulation has been shown to be associated with the poor prognosis of many cancers. Yan et al. [22] found that LINC00313 was significantly increased in both papillary thyroid carcinoma tissues and cell lines, and contributed to papillary thyroid carcinoma. Additionally, Yang et al. [23] also found that LINC00313, which is up-regulated during Kaposi's sarcoma-associated herpesvirus reactivation, interacts with human immunodeficiency virus Tat to promote endothelial cell motility. progression In this study, our results showed that LINC00313 was down-regulated in OA tissues and cells. Functionally, the overexpression of LINC00313 blocked the progression of OA, cell apoptosis and the increase of inflammatory factors in vitro. These findings indicated that the abnormal expression of LINC00313 is related to the progression of OA.

In the pathogenesis of OA, miRNAs regulate chondrocyte apoptosis and proliferation, extracellular matrix metabolism, inflammatory response, etc. [24–27]. MiR-146b can inhibit the expression of α-2-macroglobulin (A2M), increase the activity of proteolytic enzymes, promote cell apoptosis and accelerate the development of OA [28]. MiR-384-5p inhibited cell proliferation and induces apoptosis by targeting Sox9 46. MiR-127-5p targets OPN to inhibit the proliferation of chondrocytes [29]. In this study, luciferase reporter analysis showed that LINC00313 absorbed miR-525-5p. Further results showed that the overexpression of miR-525-5p reversed the effect of LINC00313 in C28/I2 cells.

GDF5, also known as cartilage-derived morphogenetic protein 1, is a member of the beta superfamily of transforming growth factors and is involved in the development, maintenance and repair of bone, cartilage and other tissues of synovial joints [30–32]. In addition, luciferase reporter analysis showed that miR-525-5p inhibited the activity of GDF5. Further rescue experiments showed that miR-525-5p inhibitor blocked the inhibitory effect of LPS on C28/I2 cells, and knockdown of GDF5 could reverse this process. These findings indicated that the miR-525-5p/GDF5 axis substantially contributes to the progression of C28/I2 cells.

In conclusion, the study revealed that LINC00313 decreased expression in OA tissues and cells. LINC00313 overexpression promoted the proliferation of OA cells by targeting the miR-525-5p/GDF5 axis and inhibited the secretion of inflammatory factors and apoptosis of OA cells.

## Data Availability

The datasets used and/or analyzed during the current study are available from the corresponding author on reasonable request.

## References

[1] Mandl LA (2019). Osteoarthritis year in review 2018: clinical. Osteoarthr Cartil.

[2] Jones IA, Togashi R, Wilson ML, Heckmann N, Vangsness CT (2019). Intra-articular treatment options for knee osteoarthritis. Nat Rev Rheumatol.

[3] Sacitharan PK (2019). Ageing and osteoarthritis. Subcell Biochem.

[4] Kun-Peng Z, Chun-Lin Z, Xiao-Long M (2017). Antisense lncRNA FOXF1-AS1 promotes migration and invasion of osteosarcoma cells through the FOXF1/MMP-2/-9 pathway. Int J Biol Sci.

[5] Sahin Y. LncRNA H19 is a potential biomarker and correlated with immune infiltration in thyroid carcinoma [published online ahead of print, 2022 Jul 9]. Clin Exp Med. 2022;10.1007/s10238-022-00853-w. 10.1007/s10238-022-00853-w 35810257

[6] Yang F, Lv S. LncRNA EPB41L4A-AS1 Regulates Cell Proliferation, Apoptosis and Metastasis in Breast Cancer [published correction appears in Ann Clin Lab Sci. 2022 May;52(3):510]. Ann Clin Lab Sci. 2022;52(1):3–11. 35181612

[7] Li Z, Dou P, Liu T, He S (2017). Application of long noncoding RNAs in osteosarcoma: biomarkers and therapeutic targets. Cell Physiol Biochem.

[8] Fiscon G, Conte F, Farina L, Paci P (2018). Network-based approaches to explore complex biological systems towards network medicine. Genes (Basel).

[9] Li M, Qiu M, Xu Y, Mao Q, Wang J, Dong G, Xia W, Yin R, Xu L (2015). Differentially expressed protein-coding genes and long noncoding RNA in early-stage lung cancer. Tumour Biol.

[10] Wu WJ, Yin H, Hu JJ, Wei XZ (2018). Long noncoding RNA LINC00313 modulates papillary thyroid cancer tumorigenesis via sponging miR-4429. Neoplasma.

[11] Zhai Y, Liu Y, Wang Z, Wang W, Zhou J, Lu J (2021). Long non-coding RNA LINC00313 accelerates cervical carcinoma progression by miR-4677-3p/CDK6 axis. Onco Targets Ther.

[12] Chen H, Chen L (2020). An integrated analysis of the competing endogenous RNA network and co-expression network revealed seven hub long non-coding RNAs in osteoarthritis. Bone Joint Res.

[13] Chen G, Liu T, Yu B, Wang B, Peng Q (2020). CircRNA-UBE2G1 regulates LPS-induced osteoarthritis through miR-373/HIF-1a axis. Cell Cycle.

[14] Xie P, Han Q, Liu D, Yao D, Lu X, Wang Z, Zuo X (2020). miR-525-5p modulates proliferation and epithelial-mesenchymal transition of glioma by targeting stat-1. Onco Targets Ther.

[15] Chen M, Liu LX (2020). MiR-525-5p repressed metastasis and anoikis resistance in cervical cancer via blocking UBE2C/ZEB1/2 signal axis. Dig Dis Sci.

[16] Francis-West PH, Abdelfattah A, Chen P, Allen C, Parish J, Ladher R, Allen S, MacPherson S, Luyten FP, Archer CW (1999). Mechanisms of GDF-5 action during skeletal development. Development.

[17] Takahara M, Harada M, Guan D, Otsuji M, Naruse T, Takagi M, Ogino T (2004). Developmental failure of phalanges in the absence of growth/differentiation factor 5. Bone.

[18] Zhang A, Ma S, Yuan L, Wu S, Liu S, Wei X, Chen L, Ma C, Zhao H (2020). Knockout of miR-21-5p alleviates cartilage matrix degradation by targeting Gdf5 in temporomandibular joint osteoarthritis. Bone Joint Res.

[19] Gebert LFR, MacRae IJ (2019). Regulation of microRNA function in animals. Nat Rev Mol Cell Biol.

[20] Wang Y, Cao L, Wang Q, Huang J, Xu S (2019). LncRNA FOXD2-AS1 induces chondrocyte proliferation through sponging miR-27a-3p in osteoarthritis. Artif Cells Nanomed Biotechnol.

[21] Zhang L, Zhang P, Sun X, Zhou L, Zhao J (2018). Biosci Rep.

[22] Yan DG, Liu N, Chao M, Tu YY, Liu WS (2019). SP1-induced upregulation of long noncoding RNA LINC00313 contributes to papillary thyroid cancer progression via the miR-422a. Eur Rev Med Pharmacol Sci.

[23] Yang WS, Lin TY, Chang L (2020). HIV-1 tat interacts with a Kaposi's Sarcoma-associated herpesvirus reactivation-upregulated antiangiogenic long noncoding RNA, LINC00313, and antagonizes its function. J Virol.

[24] Giordano L, Porta GD, Peretti GM, Maffulli N (2020). Therapeutic potential of microRNA in tendon injuries. Br Med Bull.

[25] Oliviero A, Della Porta G, Peretti GM, Maffulli N (2019). MicroRNA in osteoarthritis: physiopathology, diagnosis and therapeutic challenge. Br Med Bull.

[26] Gargano G, Oliva F, Oliviero A, Maffulli N (2022). Small interfering RNAs in the management of human rheumatoid arthritis. Br Med Bull.

[27] Gargano G, Oliviero A, Oliva F, Maffulli N (2021). Small interfering RNAs in tendon homeostasis. Br Med Bull.

[28] Liu X, Liu L, Zhang H, Shao Y, Chen Z, Feng X, Fang H, Zhao C, Pan J, Zhang H (2019). MiR-146b accelerates osteoarthritis progression by targeting alpha-2-macroglobulin. Aging (Albany NY).

[29] Tu M, Li Y, Zeng C, Deng Z, Gao S, Xiao W, Luo W, Jiang W, Li L, Lei G (2016). MicroRNA-127-5p regulates osteopontin expression and osteopontin-mediated proliferation of human chondrocytes. Sci Rep.

[30] Edwards CJ, Francis-West PH (2001). Bone morphogenetic proteins in the development and healing of synovial joints. Semin Arthritis Rheum.

[31] Harada M, Takahara M, Zhe P, Otsuji M, Iuchi Y, Takagi M, Ogino T (2007). Developmental failure of the intra-articular ligaments in mice with absence of growth differentiation factor 5. Osteoarthr Cartil.

[32] Hatakeyama Y, Tuan RS, Shum L (2004). Distinct functions of BMP4 and GDF5 in the regulation of chondrogenesis. J Cell Biochem.

